# 25.3 OLIGODENDROCYTES MEDIATE ENERGY METABOLISM ALTERATIONS IN SCHIZOPHRENIA: A PROTEOMIC STUDY

**DOI:** 10.1093/schbul/sby014.103

**Published:** 2018-04-01

**Authors:** Daniel Martins-De-Souza

**Affiliations:** University of Campinas (UNICAMP)

## Abstract

**Background:**

While comparing the proteomes and subproteomes of 8 post-mortem brain regions and cerebrospinal fluid from schizophrenia patients to controls, we consistently observed alterations in energy metabolism, cell growth and maintenance, synaptic function, and myelinization processes. Considering the nature of these analyses, it was not possible to reveal which particular cell types display such alterations. This is essential information given increasing evidence of glia cells as pivotal players in schizophrenia. With this in mind, we analyzed the proteomes and phosphoproteomes of cultured astrocytes, oligodendrocytes and neurons treated with MK-801, a NMDA-receptor antagonist which impairs glutamatergic transmission as postulated in schizophrenia. We also analyzed biochemical pathways modulated by typical and atypical antipsychotics in human oligodendrocytes. Results led us to employ induced pluripotent stem cell-derived cerebral organoids to deepen our understanding of the data. The central aim of this study is to depict which cell type(s) present proteome changes similarly to those we found in our earlier analysis of human brain tissue as well as identify key pathways for an effective antipsychotic response.

**Methods:**

Cell line cultures (astrocytes, oligodendrocytes and neurons) were treated with MK-801 and oligodendrocytes were also treated with a range of typical and atypical antipsychotics. In addition, human embryonic stem cells reprogramed from schizophrenia patients and controls fibroblasts were cultured in mTeSR1 media on Matrigel coated surface and then differentiated into cerebral organoids. All pre-clinical models here employed were submitted to state-of-the art large-scale proteomic analyses. In silico systems biology was employed to identify key pathways in the studied processes.

**Results:**

MK-801-treated astrocytes, and especially MK-801-treated oligodendrocytes displayed several proteins differentially expressed which overlapped with previous findings of schizophrenia human brains. On the other hand, MK801-treated neurons displayed very few differences in their proteome, an overlap with previous findings in human brain tissue below 10%. More interestingly, the dysregulation of glycolytic enzymes in MK801-treated oligodendrocytes are very similar to our observations in schizophrenia brain tissue, corroborating with recent findings about of the importance of oligodendrocytes in the energy status of the brain. In oligodendrocytes, antipsychotics displayed differences in translational machinery and eIF2 signaling. Findings on cerebral organoids also showed overlaps with previous postmortem data, mainly on synaptic proteins and specially energy metabolism-associated pathways.

**Discussion:**

These findings hold potential for the investigation of developmental and evolutionary features of schizophrenia brains and provides targets to be drug-screened as well as leads to the schizophrenia pathobiology.

